# A Career Crafting Training Program: Results of an Intervention Study

**DOI:** 10.3389/fpsyg.2021.664453

**Published:** 2021-05-26

**Authors:** Evelien H. van Leeuwen, Toon W. Taris, Machteld van den Heuvel, Eva Knies, Elizabeth L. J. van Rensen, Jan-Willem J. Lammers

**Affiliations:** ^1^Department of Quality and Patient Safety, University Medical Center Utrecht, Utrecht, Netherlands; ^2^Department of Strategic Human Resource Management, Utrecht University School of Governance, Utrecht, Netherlands; ^3^Department of Social, Health and Organizational Psychology, Faculty of Social and Behavioural Sciences, Utrecht University, Utrecht, Netherlands; ^4^Work and Organisational Psychology, University of Amsterdam, Amsterdam, Netherlands

**Keywords:** career crafting, career self-management, job crafting, employability, physicians, intervention study

## Abstract

This intervention study examined the effects of a career crafting training on physicians' perceptions of their job crafting behaviors, career self-management, and employability. A total of 154 physicians working in two hospitals in a large Dutch city were randomly assigned to a waitlist control group or an intervention group. Physicians in the intervention group received an accredited training on career crafting, including a mix of theory, self-reflection, and exercises. Participants developed four career crafting goals during the training, to work on in the subsequent weeks, after which a coaching conversation took place over the phone. Physicians in the control group received no intervention. A pre- and post-test 8 weeks later measured changes in job crafting and career self-management (primary outcomes) and employability (secondary outcome) of 103 physicians that completed the pre- and post-test. RM ANOVAs showed that the intervention enhanced perceptions of career self-management and job crafting behavior to decrease hindering job demands. No support was found for the effect of the intervention on other types of job crafting and employability. This study offers novel insights into how career crafting can be enhanced through training, as this is the first empirical study to examine a career crafting intervention. HR managers can use the outcomes to develop tailored career policies and career development practices.

## Introduction

Both the workforce and work environment have changed strongly during the last decades. In today's careers, organizational career support is uncommon and, in the light of increasingly diverse career needs, not a feasible option (Demerouti, 2014). Furthermore, the recent increase in the formal retirement age (Davies et al., 2017) affects employees' careers and encourages them to prolong their working lives. Together, this requires individuals to play an active role in shaping jobs and careers in line with their needs and wishes (Dubbelt et al., 2019). In response to the need for proactive employees, Akkermans and Tims (2017) introduced the *career crafting* concept, which was later defined as “individuals' proactive behavior aimed at optimizing career outcomes through improving person-career fit” (De Vos et al., 2019, p. 129). Proactively taking responsibility for one's career is assumed to be important for safeguarding the sustainability of one's career over time. It is the dynamic process of adapting to one's career-related context and proactively working toward a fulfilling career. This may contribute to an individual's employability (De Vos et al., 2019). Employability, which is defined as the ability and willingness to work and to continue working in the current profession until retirement age (e.g., Oude Hengel et al., 2012), is currently challenged by an increasingly complex and changing career landscape (Frenk et al., 2010). This complexity results in high job demands and requires employees to acquire new knowledge and skills. Attention for career crafting behavior of employees, aimed at optimizing employability, is therefore especially important today.

Career crafting is a new area of research. In this study, we build on the existing conceptualizations of career crafting (e.g., De Vos et al., 2019; Tims and Akkermans, 2020) by further clarifying the types of proactive behavior that are undertaken when individuals craft their career. Since a career consists of past, present, as well as future work experiences (Arthur et al., 1989), examining careers empirically necessarily implies a temporal focus. We propose to refine the conceptualization of career crafting to “individuals' proactive behaviors targeted at changing both their present as well as their future work situation, with the ultimate aim to optimize career outcomes.” Such a refined conceptualization is necessary in order to actively target individuals' specific behaviors via an intervention. Therefore, in this intervention study, we operationalize career crafting as an umbrella concept, consisting of two types of proactive behavior, including (i) actions focused on optimizing the current job and work environment via job crafting and (ii) proactive behavior focused on the longer-term career (i.e., career self-management behavior; Van Leeuwen et al., 2020).

Despite the importance of this novel area of research, to date, few empirical studies have examined career crafting directly. These studies have either examined *job crafting behavior* (e.g., Rudolph et al., 2017; Oprea et al., 2019; Knight et al., 2021), referring to the “self-initiated behaviors that employees take to shape and change their jobs” (Zhang and Parker, 2018, p. 126), or *career behavior* interventions (e.g., Koen et al., 2012; Spurk et al., 2015). These two streams of literature have developed in isolation (Tims and Akkermans, 2020). Although both streams of literature make important contributions to a better understanding of proactive behavior at work, job crafting and career behavior interventions target different aspects (either current job design or longer-term career). Career research would benefit from adding the notion of *career crafting* (Tims and Akkermans, 2020).

Besides, there is a lack of knowledge on how to enhance employability as this has not been studied in an intervention setting yet as far as we are aware. The importance of employability is widely acknowledged (e.g., Fleuren et al., 2016) and growing in the current COVID-19 crisis. Numerous studies have examined the antecedents and/or outcomes of employability (e.g., Van Dam, 2004; Van Harten, 2016; Le Blanc et al., 2019). While proactive behaviors, like job and career crafting, are suggested to enhance the sustainability of one's career (employability) (De Vos et al., 2019; Plomp et al., 2019), empirical evidence supporting this assumption lacks. Our study contributes by first testing whether a newly developed intervention can trigger career crafting behaviors in participants, and secondly, by testing whether the intervention can positively impact employability perceptions, either directly or indirectly through career crafting.

Furthermore, we examine an occupational group that is relatively understudied when it comes to interventions that can boost proactive career behavior such as career crafting. Physicians are such a group. Many studies that examine physicians focus on outcome measures such as well-being (e.g., West et al., 2014), burnout (e.g., Goodman and Schorling, 2012; West et al., 2016), or engagement (e.g., Shanafelt and Noseworthy, 2017). The findings of these studies show a high level of distress among physicians and a high prevalence of burnout. This can be related to reduced employability among physicians, referring to a lack of ability and/or willingness to meet the high demands of their work tasks. This emphasizes the importance of finding ways that help physicians to cope with their work environment and remain employable. Developing proactive work behaviors, such as career crafting, are likely to play a crucial role in this. However, these behaviors are understudied among physicians (with the notable exception of Gordon et al., 2018, who studied job crafting behavior among physicians). Furthermore, more attention for the employability of physicians is warranted, as studies into employability mainly focus on employees in general. By explicitly focusing on career crafting behavior and employability of physicians, this study responds to recent calls for more contextualized research (Akkermans and Kubasch, 2017; Knies et al., 2018).

Responding to these knowledge gaps, this intervention study examines the effects of a career crafting training on physicians' career crafting behavior and employability. The central question addressed in this study is: What are the effects of a group-level training on physicians' career crafting behavior (i.e., job crafting behavior and career self-management) and employability? This study contributes to the literature on career crafting by empirically examining the effects of a recently developed career crafting intervention in which systematic development is elaboratively described in Van Leeuwen et al. (2020). In doing so, this study aims to integrate the fields on job crafting and career behavior conceptually and empirically. In addition, the intervention design offers an important methodological contribution to the field of employability, where this is one of the first studies examining how employability may be enhanced via an intervention.

## Theoretical Background and Hypotheses

### Career Crafting Conceptualized

Career crafting is a relatively new concept, introduced by Akkermans and Tims (2017) and further conceptualized by De Vos et al. (2019) and Tims and Akkermans (2020). According to De Vos et al. (2019), career crafting refers to proactive behavior aimed at optimizing career outcomes. Tims and Akkermans (2020) identified that career crafting includes two elements: studying (1) the shaping of individual *jobs* and (2) the series of jobs or roles that comprise their *career journeys*. This study builds on these conceptualizations and further specifies individuals' specific proactive behaviors, which is necessary to examine career crafting empirically. Proactively crafting one's individual job, the short-term aspect, is represented in this study by job crafting. Proactively crafting the series of jobs that comprise a career, the long-term aspect, is represented here as career self-management.

#### Job Crafting

There are different perspectives on job crafting. Two dominant perspectives are introduced by Wrzesniewski and Dutton (2001) and Tims et al. (2012). In their seminal 2001 paper, Wrzesniewski and Dutton defined job crafting as the physical and cognitive changes individuals make in the task or relational boundaries of their work (p. 179). One of the elements in their definition of job crafting is adapting job tasks so that they match employees' personal resources. Kooij et al. (2017) integrated this in their operationalization of job crafting by defining this concept as employees' initiative to adapt their job to their personal strengths and interests. They distinguish between job crafting toward *strengths* and job crafting toward *interests* defined as the self-initiated changes that individuals make in the task boundaries of their work to make better use of their *strengths* and to make it better fit their *interests* (Kooij et al., 2017, p. 972).

A second popular perspective on job crafting starts from the job demands-resources model and argues that job crafting can be directed toward changing job demands and/or job resources (Tims et al., 2012). This approach proposes that job characteristics can be classified into either of two categories: job demands or job resources. Job demands refer to job aspects that require sustained physical and/or psychological effort or skills (e.g., high workload, physical demanding work). Being exposed to job demands for extended periods of time can lead to fatigue and strain responses. Job resources refer to job aspects that are functional in achieving work goals, reduce job demands and the associated negative effects, and stimulate personal development (e.g., autonomy, social support; Tims et al., 2012). Employees can proactively lower their job demands when they perceive that their demands have become overwhelming. Employees may also increase job resources, for instance, by actively looking for support.

In this study, we combine the perspectives of job crafting toward personal resources (Wrzesniewski and Dutton, 2001; Kooij et al., 2017) with the perspective of job crafting toward job characteristics (Tims et al., 2012). This is relevant since these two perspectives focus on different areas of job crafting which can both play a role in physicians' work.

#### Career Self-Management

Besides crafting their current job, employees may attempt to proactively manage their career. A variety of concepts has been used to refer to proactive career behaviors, such as career engagement (Hirschi et al., 2014), career self-directedness (De Vos and Segers, 2013), and career self-management (e.g., Kossek et al., 1998). In this study, we focus on career self-management since it emphasizes the role of the individual employee and their proactive behavior. Career self-management refers to the proactive actions that employees take to influence their career experiences (cf. De Vos and Soens, 2008; Hirschi et al., 2014). The role of the employee is stressed in items measuring career self-management, by focusing on their proactive behavior including general career behaviors, focused on the design of the professional future, and specific career behaviors: career planning, career self-exploration, environmental career exploration, networking, voluntary human capital and skill development, and positioning behavior (Hirschi et al., 2014).

#### Differences Between Job Crafting, Career Self-Management, and Career Crafting

Job crafting and career self-management both focus on proactive work behaviors initiated by the employee. These concepts differ in the subject of these behaviors. While job crafting is concerned with current work design, career self-management primarily focuses on changing elements in the series of jobs or roles that comprise careers (Tims and Akkermans, 2020). A second difference is the scope of job crafting and career self-management, which is narrower in career self-management, which includes general as well as specific behaviors (e.g., career planning or networking; Tims and Akkermans, 2020). Career intervention studies often mainly focus on specific career competencies, such as networking (Spurk et al., 2015), career adaptability (Koen et al., 2012), or on career management preparedness (Vuori et al., 2012), rather than on training proactive career behavior *in general* (i.e., including multiple career behaviors) as the career crafting intervention aims to do (see Raabe et al., 2007, for a notable exception). Job crafting has a broader scope than career intervention studies as it refers to proactive behavior in relation to job characteristics (job demands and job resources) and personal characteristics in general (strengths and interests).

Paying more attention to career crafting is essential as employees have new and more diverse career needs (Demerouti, 2014; Dubbelt et al., 2019), making it important that they proactively shape their careers in order to meet their own career needs. Also, employability is challenged in an aging workforce due to the recent increase in the formal retirement age (White et al., 2018). These developments may be even more important for specific occupational groups. For physicians, the increase in the retirement age might further challenge their ability to work until the new official retirement age, set at 67 years in the Netherlands, as their work is already physically and emotionally demanding. Furthermore, their career trajectories are different from other employees. Physicians usually work in the same job throughout their entire career. As opportunities to change occupations are limited, staying employable within their profession is therefore especially important (Freidson, 1994). Moreover, physicians describe their work as highly individualistic, with poor mentorship, little support (Levine et al., 2011), and weak support structures (West et al., 2018). This non-supportive environment raises the importance of physicians taking responsibility for their work and careers. Physicians can make several choices in their career design. They can decide to further specialize in a certain area of their work. Besides making changes in their clinical work, they can also become active in other areas such as education, research, or leadership, where they can follow certain training programs for. Other possibilities are becoming members of certain committees, within the hospital, or within their professional associations, such as committees about the quality and safety of patient care. Taking responsibility for career design is suggested to be important to create a sustainable career (De Vos et al., 2019). In the present study, we therefore introduce and examine a training that aims to increase physicians' attempts at career crafting.

### The Career Crafting Training

The intervention tested in the present study is a group-level training combining theory, practical exercises, and action planning. It was developed using a systematic intervention development protocol (Van Leeuwen et al., 2020). The intervention is expected to enhance job crafting and career self-management. This hypothesized relationship can be explained by the social-cognitive theory (Bandura, 1991) and the theory of planned behavior (Ajzen, 1991). Bandura's (1991) social-cognitive theory emphasizes three self-regulative mechanisms that motivate and regulate behaviors: self-monitoring, judgment of one's behavior, and affective self-reaction (Bandura, 1991). These three aspects are present in this intervention study. Self-monitoring refers to self-assessing one's current work situation, personal ambition, and performance in various exercises. For instance, physicians may reflect on what aspects of their job they are proud of and which aspects of their job they find unsatisfactory. In the judgment process, people reflect on their own behavior and compare it to their personal standards and to others. Social comparison is stimulated in this training through the exchange of experiences with peers. Affective self-reaction refers to the evaluation of behavior. This is likely to happen in the period after the training, in which participants put their personal development plan into practice. After this part, a coaching conversation took place over the phone in which their experiences were discussed.

The presence of these three aspects underlined by social-cognitive theory in the career crafting intervention results in the expectation that the intervention will ultimately lead to higher levels of job crafting and career self-management behavior. Specifically, the intervention should result in a more favorable attitude toward these career crafting behaviors as well as the perception that one is in control of such behaviors. Based on Ajzen's (1991) theory of planned behavior, we expect that this will lead to a higher intention to engage in these behaviors, which should in turn stimulate actual career crafting behavior. In this intervention, physicians develop four career crafting goals. Since these goals are self-set, and as physicians have a high level of job autonomy, their perceived behavioral control is likely to be high. Furthermore, physicians are encouraged to develop meaningful and realistic career goals. This will result in goals that are valuable to them, which is likely to result in physicians having a favorable attitude toward the behaviors needed to achieve these goals in the future. Taken together, this results in the following hypotheses:

*Hypothesis 1:* At Time 2, physicians in the intervention group will report higher levels of (a) job crafting toward strengths, (b) job crafting toward interests, (c) job crafting to decrease hindering job demands, and (d) job crafting to increase social job resources, compared to Time 1 as well as to a control group.

*Hypothesis 2:* At Time 2, physicians in the intervention group will report higher levels of career self-management behavior, compared to Time 1 as well as to a control group.

### The Influence of Career Crafting on Employability

There are different conceptualizations of employability. Forrier et al. (2015) divide these in three categories: (1) the personal strength perspective, focusing on employees' competences or abilities (e.g., Van der Heijde and Van Der Heijden, 2006) and attitudes or willingness to work (e.g., Van Dam, 2004); (2) an individuals' chance of finding employment; and (3) the realization of job transitions. The first perspective best fits the situation of physicians, who commonly work in the same profession until retirement, due to the long period of education that they followed and due to their high level of specialization. Job transitions are therefore unlikely, and finding employment is less relevant since physicians often remain in the same job throughout their careers. Instead, staying able and willing to continue working in the current profession is highly relevant for them. Employability is therefore operationalized in this study as physician's *ability* and *willingness* to work and to continue working in the current profession until retirement age (based on De Vos et al., 2011; Oude Hengel et al., 2012; Froehlich et al., 2014; Van Harten, 2016).

This perspective on employability is in line with this study's focus on the proactivity of employees. In order to continue to work, someone's expertise and skills or ability to continue to work are important, which is highlighted in studies on employability (Clarke, 2007). In addition, the motivation or willingness to continue to work is essential, which is a second aspect underlined in studies on employability (Rothwell and Arnold, 2007).

Career crafting is considered an important individual behavior aimed at safeguarding the sustainability of one's career over time (De Vos et al., 2019). Although not yet supported by empirical evidence, this suggests a positive causal relationship between career crafting and employability. Theoretically, this makes sense since job crafting to seek resources is likely to enlarge an employee's pool of resources (Dubbelt et al., 2019), and job crafting toward strengths aims to increase the fit between abilities and job content. This is assumed to enhance feelings of competence, resulting in the perception of being better *able* to continue to work. Job crafting to decrease job demands and job crafting toward interests may enhance the *willingness* to continue to work. Assuming that attempts to reduce hindering job demands are successful, this would result in a job with fewer hindering aspects (Dubbelt et al., 2019). Besides, job crafting toward interests aims to increase the alignment of a persons' interests and his job content. This should create positive perceptions on the willingness to continue to work.

Furthermore, career self-management behavior is likely to positively affect employability as it may help employees to keep up with the fast-changing environment (Tims and Akkermans, 2020), which might be beneficial for their ability to continue to work. Also, by engaging in career self-management behavior, employees invest in their personal development. This should result in finding greater value in one's work (Tims and Akkermans, 2020), since the actions that employees will perform are based on their own interests. This is likely to create positive feelings toward their willingness to continue to work.

The intervention may also directly influence physicians' employability. In the training, attention is paid to the challenges that physicians experience in their work, which can be examples of challenges around employability. Discussing these challenges and receiving advice from their peers on how to enhance their employability may result in perceptions of being better able and willing to continue to work. Moreover, the time spent in the training on employability may have enhanced physicians' confidence in their own employability. This could result in a direct relationship between the intervention and perceptions of employability. In this study, we explore whether the intervention enhances employability directly, or whether there is an indirect relationship between the intervention and employability via career crafting behavior. This leads to the following hypothesis:

*Hypothesis 3:* At Time 2, physicians in the intervention group will report higher levels of (a) physical ability to continue to work, (b) mental ability to continue to work, and (c) willingness to continue to work, compared to Time 1 as well as to a control group.

## Method

### Procedure and Participants

Physicians in this study participated in an intervention study. Assuming a two-tailed significance level of 5% and a power level of 95%, our calculations indicated that ~120 physicians (60 in each group) were needed to detect effect sizes (*f*) of 0.25 and over. This calculation was based on the effect sizes obtained in a previous job crafting intervention study (Gordon et al., 2018) and a career coaching intervention study (Spurk et al., 2015). With 154 physicians willing to participate in this intervention, we therefore had enough power at the start of this study to detect possible effects.

Physicians of two Dutch hospitals (a general hospital and an academic hospital) were invited to this study in presentations held at their departments. The University Medical Center Utrecht confirmed that formal ethical approval was not required as this study falls outside the scope of the Dutch Law on Medical Research (WMO) (METC 2019, 19/109/C). Physicians received an invitation to the survey by e-mail. The survey started with a cover letter, informing them about the content and goal of the study, with the assurance that responses would be kept confidential and that participation was voluntary. Participants provided informed consent before moving on to the survey items.

### Intervention

A parallel group trial design was used in this study. Physicians were randomly assigned to either the intervention or the waitlist control group. After randomization, physicians completed a pretest, which was a survey measuring perceptions on job crafting behavior, career self-management, and employability (see below for details). Subsequently, physicians in the intervention group received a 4-h accredited group training on career crafting. The first 2-h of the training were focused on job crafting, and the latter two were focused on career self-management. Attention was paid during the training to theory about these concepts. Also, examples of how career crafting behavior of physicians could look like were discussed. Physicians were given a handout with possibilities for engaging in career crafting behavior that were available in the organization where they worked. During the intervention, plenty of time was spend on self-reflection, through physicians sharing experiences in the group. Diverse exercises were done during the training, including a mindfulness exercise. Additionally, attention was paid to goal-setting as physicians should leave the training with four self-set goals, on which they were planning to work on in the subsequent 4 weeks. At the end of these weeks, a coaching conversation of around 15 min took place over the phone. These micro-coaching sessions focused on goal attainment given the content of the personal career crafting plans, as well as potential future actions. The aim of these coaching sessions was three-fold: first, to incentivize training transfer, i.e., investing time to work on their goals after the training; second, to allow participants to reflect on their progress, what obstacles they encountered, and what went well; and third, to support them in formulating any further follow-up actions based on their experiences so far. Three weeks after the coaching conversation, that is, 8 weeks after the pretest, a post-test was completed by all participants, again measuring job crafting, career self-management, and employability.

This intervention was systematically developed following six steps of Bartholomew et al.'s (2016) Intervention Mapping protocol: (1) needs assessment, (2) definition of program objectives, (3) methods and practical applications, (4) intervention program development and pilot test, (5) adoption and implementation, and (6) evaluation. In short, this means that physicians' needs and experiences with job crafting and career self-management were explored in 40 exploratory interviews. Subsequently, three program objectives were developed: enhancing job crafting, career self-management, and employability. Various methods were used in the training to achieve these aims, such as knowledge transfer, modeling, discussion, and elaboration. Self-reflection and imagery were further used to enhance participants' awareness of their work goals, values, and interests. Goal-setting, feedback, and public commitment by sharing goals were used to enhance self-efficacy. A pilot version of the training was tested among five physicians. This resulted in several adjustments in the allocation of time and wording to adjust the content better to the perspectives of physicians. For an elaborate version of how this intervention was developed, we refer to Van Leeuwen et al. (2020).

Block randomization was done with hospital type (an academic and a general hospital) as a blocking factor using the randomizer function in Microsoft Excel. Block randomization increases the probability of equally dividing physicians in one hospital to the control or intervention group. This is important as physicians working in these two hospitals are expected to differ on characteristics that may affect career crafting behavior, such as type of contract (being employed by the hospital or working as independently established) and degree of specialization.

**Figure 1:**
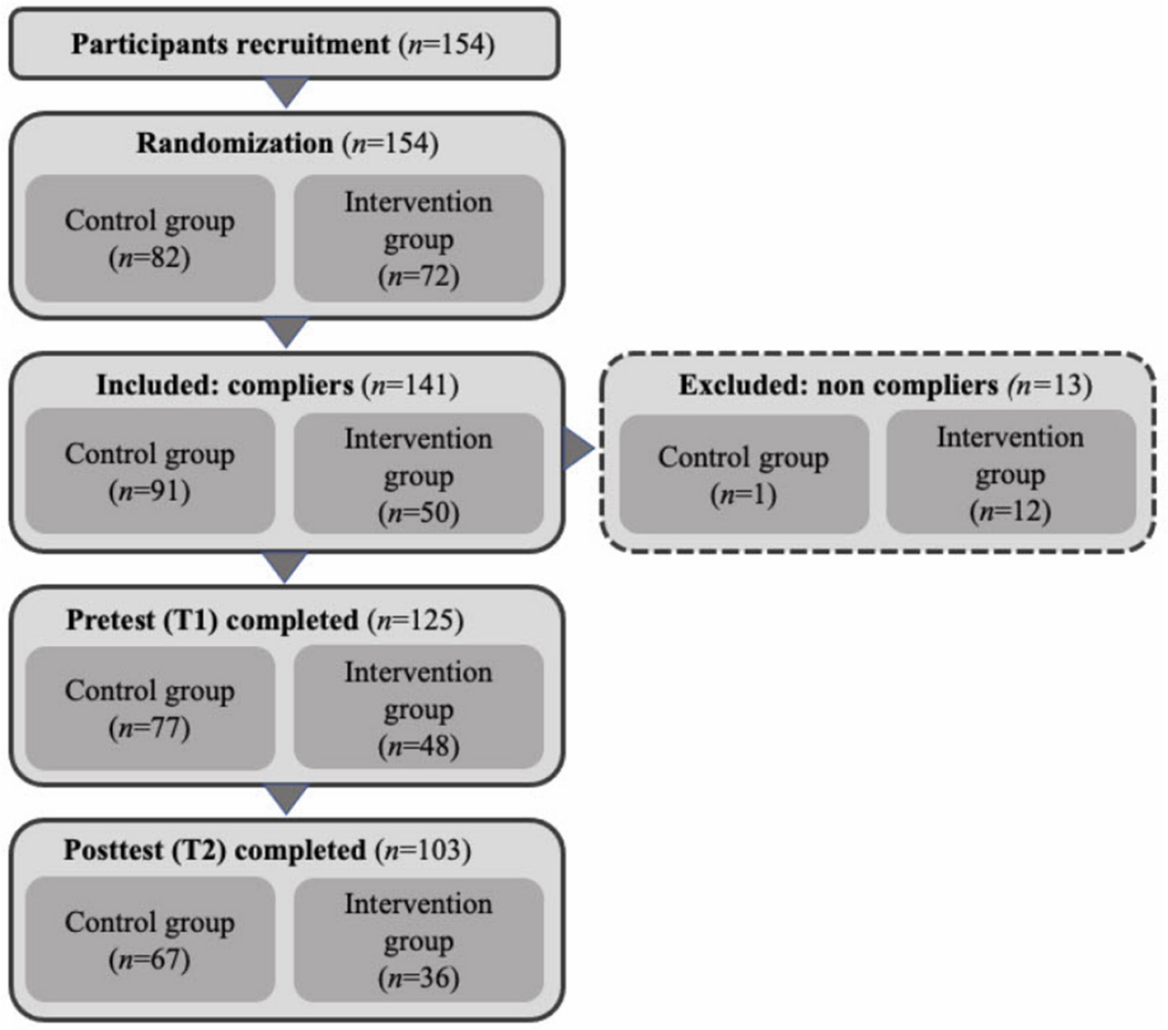
Participants flow diagram.

Figure 1 describes the process of participant recruitment. Physicians were blind to the group (control or intervention) that they were allocated to. Ten physicians did not adhere to the randomization. Correspondence via e-mail and in follow-up interviews indicated that the majority of these physicians did not comply with the randomization because of random factors that seemed unlikely to influence the randomization: one physician had a parent day at her child's school, three physicians were on pregnancy leave, one physician had a holiday planned, two physicians were abroad, one physician had to work, and two physicians were ill. These physicians were allowed to switch dates, resulting in some physicians switching from the intervention to the control group (*n* = 10)^1^. No physicians switched from the control to the intervention group. Apart from these 10 physicians, some physicians did not adhere to the study protocol, meaning that they had been assigned to the intervention group but did not follow the training (*n* = 12), or they were assigned to the control group and withdrew from the study (*n* = 1). These 13 physicians, who were not subjected to the intended intervention, were excluded from the study. In the end, the control group consisted of 91 physicians and the intervention group of 50 physicians.

Several tests were done to examine whether the physicians in the control group (*n* = 91) differed on any of the study control variables (gender, age, employment contract, hours worked according to contract, functional tenure, and organizational tenure) from physicians in the intervention group (*n* = 50). A multivariate test was not significant [*F*_(6, 105)_ = 2.021, *p* = 0.069]. Univariate follow-up tests revealed no significant differences between the groups either, all *p*s > 0.235. Thus, physicians in the waitlist control group and intervention group did not differ on any of the study control variables.

Possible contamination between physicians in the control or intervention group was reduced by asking participants not to discuss their experiences of the training with others than their colleagues who were present in the same training session. Furthermore, as participating physicians worked across a variety of departments in the hospitals, the chance of contamination is rather limited.

### Outcome Measures

Job crafting behavior and career self-management behavior were primary and proximal outcomes in this study, and employability was a secondary and distal outcome.

#### Job Crafting

Perceptions of job crafting behavior were measured using two scales. Job crafting toward strengths was measured with four items (Kooij et al., 2017), including “I organize my work in such a way that it matches with my strengths” (α_T1_ = 0.83; α_T2_ = 0.83). Job crafting toward interests was measured with five items (Kooij et al., 2017), including “I actively look for tasks that match my own interests” (α_T1_ = 0.86; α_T2_ = 0.85). Job crafting to increase social job resources was measured with five items (Tims et al., 2012), including “I ask others for feedback on my job performance” (α_T1_ = 0.72; α_T2_ = 0.68). Job crafting to decrease hindering job demands was measured with six items (Tims et al., 2012), including “I organize my work in such a way to make sure that I do not have to concentrate for too long a period at once” (α_T1_ = 0.73; α_T2_ = 0.79). All responses were given on a five-point scale (1 = never, 5 = very often).

#### Career Self-Management

Career self-management was measured with nine items of the validated career engagement scale assessing general career behaviors, career planning, career self-exploration, environmental career exploration, networking, voluntary human capital/skill development, and positioning behavior (Hirschi et al., 2014). For example, “To what extent have you, in the past 8 weeks, developed plans and goals for your future career?” (α_T1_ = 0.93.; α_T2_ = 0.92). All answers were given on a five-point scale (1 = never, 5 = very often).

#### Employability

Perceptions on employability were assessed using the items from Oude Hengel et al. (2012). Three items ask directly for perceptions on the physical ability, the mental ability, and willingness to continue to work until the retirement age in the current profession. An example item is: “I am physically able to continue to work until the age of 67 in my current profession” (1 = totally disagree, 5 = totally agree).

### Analysis

The data were analyzed with two-way repeated-measures ANOVAs (RM ANOVAs). Time (T1 vs. T2) served as a within-subject factor, and Group (control vs. intervention group) and Type (type of job crafting and indicators of employability) as between-subject factors.

#### Non-response Analyses

At T1, 125 participants completed the pretest (77 in the control group and 48 in the intervention group). At T2, 103 participants completed the post-test (67 in the control group and 36 in the intervention group). The response rate of the control group was 87%, and that of the intervention group was 75% [$\chi_{\left(1,125\right)}^{2}$ = 2.94, *p* = 0.09].

A multivariate analysis of variance indicated that participants who did not complete the post-test (*n* = 22) did not significantly differ from participants who completed the post-test (*n* = 103) on the control variables (gender, age, employment contract, hours worked according to contract, functional tenure, organizational tenure, job crafting at T1, career self-management at T1, and employability at T1), *F*_(13, 59)_ = 1.076, *p* = 0.397. Univariate follow-up tests revealed no significant differences between the groups either, all *p*s > 0.064.

Pairwise deletion was used in RM ANOVAs for participants who did not complete the pretest (*n* = 16) or post-test (*n* = 22). This resulted in 103 physicians who were included in the analyses. Table 1 presents the means, standard deviations, and correlations of the non-dichotomous study variables.

**Table 1:** Means, standard deviations, and Pearson correlations of the main study variables at T1 and T2.

	**Intervention group**		**Control group**															
	***M***	***SD***	***M***	***SD***	**1**	**2**	**3**	**4**	**5**	**6**	**7**	**8**	**9**	**10**	**11**	**12**	**13**	**14**
1. JC-toward strengths at T1	3.51	0.75	3.44	0.83	1	0.49^**^	0.51^**^	0.30	0.05	0.29	−0.07	−0.14	0.08	0.16	0.17	0.35^*^	0.11	0.33
2. JC-toward strengths at T2	3.45	0.65	3.48	0.77	0.71^**^	1	0.31	0.50^**^	0.13	−0.11	0.19	0.24	0.10	0.13	0.27	0.37^*^	0.47^**^	0.27
3. JC-toward interests at T1	2.98	0.70	3.13	0.80	0.70^**^	0.62^**^	1	0.37^*^	−0.12	0.32	−0.01	−0.05	0.16	0.02	0.20	0.25	0.22	0.38^*^
4. JC-toward interests at T2	3.14	0.75	3.15	0.84	0.49^**^	0.68^**^	0.63^**^	1	0.37	0.05	0.26	0.18	0.43^*^	0.33	0.32	0.16	0.37^*^	0.26
5. JC-to decrease hindering job demands T1	1.61	0.50	1.76	0.59	0.00	0.13	−0.04	0.12	1	0.57^**^	0.13	0.08	0.19	0.07	0.03	−0.22	0.07	−0.12
6. JC-to decrease hindering job demands T2	1.94	0.51	1.67	0.66	0.12	0.26^*^	0.19	0.30^*^	0.47^**^	1	0.22	−0.04	0.07	−0.06	−0.19	−0.22	−0.04	−0.06
7. JC-to increase social job resources T1	2.75	0.56	2.89	0.70	0.43^**^	0.48^**^	0.55^**^	0.41^**^	0.05	0.22	1	0.44^**^	0.09	0.07	0.07	0.02	0.31^*^	0.14
8. JC-to increase social job resources T2	2.87	0.74	2.81	0.62	0.30^*^	0.41^**^	0.34^**^	0.47^**^	0.09	0.27^*^	0.59^**^	1	0.21	0.24	0.31	0.07	0.20	0.30
9. Career self-management T1	2.24	0.71	2.36	0.98	0.41^**^	0.28^*^	0.41^**^	0.47^**^	0.14	0.41^**^	0.30^*^	0.18	1	0.73^**^	0.03	−0.14	−0.08	−0.10
10. Career self-management T2	2.61	0.77	2.42	0.94	0.29^*^	0.45^**^	0.42^**^	0.55^**^	0.00	0.49^**^	0.37^**^	0.36^**^	0.67^**^	1	0.21	0.08	−0.07	−0.07
11. Ability to continue to work at T1	3.89	0.89	3.78	0.94	0.33^**^	0.46^**^	0.43^**^	0.42^**^	0.02	0.09	0.20	0.08	0.25^*^	0.16	1	0.31	0.59^**^	0.60^**^
12. Ability to continue to work at T2	4.03	0.64	3.88	0.90	0.32^*^	0.46^**^	0.44^**^	0.43^**^	0.11	0.04	0.13	0.18	0.14	0.16	0.78^**^	1	0.39^*^	0.51^**^
13. Willingness to continue to work at T1	3.28	1.16	3.11	1.15	0.24^*^	0.22	0.30^*^	0.21	0.01	0.04	0.12	0.03	0.21	−0.04	0.68^**^	0.54^**^	1	0.74^**^
14. Willingness to continue to work at T2	3.13	1.07	3.12	1.08	0.08	0.22	0.16	0.21	−0.08	0.05	0.02	0.04	0.14	−0.03	0.64^**^	0.53^**^	0.90^**^	1

*Results for the control group (n = 77) are shown under the diagonal; results for the intervention group (n = 48) are above the diagonal*.

* *Correlation is significant at the 0.05 level (two-tailed)*.

** *Correlation is significant at the 0.01 level (two-tailed)*.

### Process of the Intervention

Besides an effect evaluation, a process evaluation offers insight into the factors that may have influenced the effectiveness of the intervention, which helps to understand why (parts of) an intervention results in a certain outcome (Nielsen and Randall, 2013). Three commonly examined dimensions in process evaluations are (1) context, (2) implementation process, and (3) participant attitudes toward implementation of the intervention (Nielsen and Randall, 2013; Moore et al., 2015). These were examined in this study with qualitative methods (by asking for physicians' experiences in the coaching conversation and in open-ended survey questions) and quantitative methods (answers to survey questions).

Physicians mentioned several barriers and facilitators within the context that affected the implementation of their career crafting goals in the weeks after the training. A high workload, being sick, having a holiday, or being on a congress were mentioned as barriers. Facilitators of working on their career crafting goals that were raised included self-set reminders that physicians put in their agenda during the training, reminders that physicians received from the researchers by e-mail, and the coaching conversation. No other sudden events that affected their opportunity to work on their career crafting goals were raised.

Regarding the implementation process, training sessions were similar in duration and content. There were two unplanned differences between the training sessions. Group sizes varied from 4 to 10 physicians due to cancellations, and the type of issues that physicians raised during group discussion differed between training sessions. This was the result of asking physicians to raise personal issues.

Furthermore, participant attitudes toward the implemented intervention were assessed. Physicians reported that the training atmosphere was supportive (*M* = 4.42, *SD* = 0.55) and felt safe (*M* = 4.47, *SD* = 0.69) (scale 1–5). Physicians were also positive about the training in general (*M* = 7.89, *SD* = 0.92) and about the trainer (*M* = 8.11, *SD* = 0.69) (scale 1–10). Regarding participation, all physicians followed a 4-h training and developed four self-set career crafting goals. A few physicians left some minutes before the end of the training because of personal reasons. In addition, physicians worked on average 4-h on two or three career crafting goals.

## Results

### Effect of the Intervention on Job Crafting

A series of four two-way Time (pretest at T1 vs. post-test at T2) × Group (control vs. intervention group) × Type (job crafting toward strengths, toward interests, to increase social job resources, and to decrease hindering job demands) RM ANOVAs analyzed the effects of the training on job crafting. While the main effect of Time [*F*_(1, 61)_ = 3.570, *p* = 0.064], the Type × Group interaction [*F*_(3, 59)_ = 0.365, *p* = 0.779], and the Time × Type × Group interaction [*F*_(3, 59)_ = 1.336, *p* = 0.271] were not significant, the main effect of Type [*F*_(3, 59)_ = 93.327, *p* ≤ 0.001, partial η^2^ = 0.826], the Time × Group interaction [*F*_(1, 61)_ = 4.119, *p* = 0.047, partial η^2^ = 0.063], and the Time × Type interaction [*F*_(3, 59)_ = 3.171, *p* = 0.031, partial η^2^ = 0.139] were significant.

As the main effect of Type of job crafting was significant, this effect was further probed by looking at the different elements of job crafting. Table 2 shows that for job crafting toward strengths, job crafting toward interests, and job crafting to increase social resources, no significant effects were obtained in the intervention and control group. Conversely, a significant Time × Group interaction effect was found for job crafting to decrease hindering job demands [*F*_(1, 63)_ = 5.348, *p* = 0.024, partial η^2^ = 0.081]. This entails that job crafting to decrease hindering job demands increased significantly from T1 (*M* = 1.58, *SD* = 0.50) to T2 (*M* = 1.88, *SD* = 0.54) in the intervention group [*F*_(1, 22)_ = 9.625, *p* = 0.005, partial η^2^ = 0.304], compared to a non-significant change from T1 (*M* = 1.76, *SD* = 0.62) to T2 (*M* = 1.68, *SD* = 0.69) in the control group [*F*_(1, 41)_ = 0.468, *p* = 0.498].

**Table 2:** Results of RM ANOVAs job crafting (*n*_control_ = 50; *n*_intervention_ = 38).

	**Intervention group**				**Control group**			
	***M***		**RM ANOVA**		***M***		**RM ANOVA**	
**Job crafting**	**T1**	**T2**	***F-*values**	**η^2^**	**T1**	**T2**	***F-*values**	**η^2^**
.. toward strengths	3.51	3.43	*F*_(1, 34)_ = 0.466, *p* = 0.499	0.014	3.48	3.48	*F*_(1, 61)_ = 0, *p* = 1.0	0
.. toward interests	3.03	3.14	*F*_(1, 34)_ = 0.660, *p* = 0.422	0.019	3.14	3.19	*F*_(1, 62)_ = 0.307, *p* = 0.582	0.005
.. to decrease hindering job demands	1.58	1.88	*F*_(1, 22)_ = 9.625, *p* = 0.005^**^	0.304	1.76	1.68	*F*_(1, 41)_ = 0.468, *p* = 0.498	0.011
.. to increase social job resources	2.78	2.86	*F*_(1, 35)_ = 0.467, *p* = 0.499	0.013	2.85	2.80	*F*_(1, 64)_ = 0.388, *p* = 0.536	0.006

** *RM ANOVA significant at the 0.01 level (two-tailed)*.

Altogether, these findings show that the career crafting intervention enhanced physicians' job crafting behavior to decrease hindering job demands from T1 to T2 (supporting hypothesis 1c), whereas no support was found for a positive relationship between the intervention and other types of job crafting (hypotheses 1a, 1b, and 1d rejected).

### Effect of the Intervention on Career Self-Management

A two-way Time (pretest at T1 vs. post-test at T2) × Group (control vs. intervention group) RM ANOVA indicated a significant difference in perceptions on career self-management for physicians in the control and intervention group from T1 to T2 [*F*_(1, 92)_ = 4.585, *p* = 0.035, partial η^2^ = 0.047]. Perceptions on career self-management increased significantly from T1 (*M* = 2.24, *SD* = 0.75) to T2 (*M* = 2.61, *SD* = 0.75) in the intervention group [*F*_(1, 32)_ = 14.491, *p* = 0.001, partial η^2^ = 0.312], compared to a non-significant change from T1 (*M* = 2.34, *SD* = 0.91) to T2 (*M* = 2.39, *SD* = 0.92) in the control group [*F*_(1, 60)_ = 0.269, *p* = 0.606].

This shows that physicians' career self-management behavior increased from T1 to T2, caused by the career crafting intervention (hypothesis 2 supported).

### Effect of the Intervention on Employability

A two-way Time (pretest at T1 vs. post-test at T2) × Group (control vs. intervention group) × Type (physical ability, mental ability, and willingness to continue to work) RM ANOVA showed non-significant main effects for Time [*F*_(1, 89)_ = 1.092, *p* = 0.299], Time × Group interaction [*F*_(1, 89)_ = 2.444, *p* = 0.122], Type × Group interaction [*F*_(2, 88)_ = 0.189, *p* = 0.828], Time × Group interaction [*F*_(2, 88)_ = 0.431, *p* = 0.651], and Time × Type × Group interaction [*F*_(2, 88)_ = 0.359, *p* = 0.700]. Only a significant main effect for Type was found [*F*_(2, 88)_ = 24.231, *p* < 0.001]. This indicates that outcomes of the analysis differed among the different indicators of employability (the physical ability, mental ability, and willingness to continue to work). These tests show no significant changes from T1 to T2 in the control and intervention groups.

*Post-hoc*, we examined the indirect effect of the intervention on employability through job crafting behavior. This indirect effect was not significant for any of the four job crafting dimensions: job crafting toward strengths (*b* = 0.03, 95% CI: −0.09 to 0.16, *ns*), toward interests (*b* = 0.01, 95% CI: −0.11 to 0.13, *ns*), to decrease hindering job demands (*b* = −0.00, 95% CI: −0.08 to 0.09, *ns*), and to increase social job resources (*b* = −0.02, 95% CI: −0.09 to 0.05, *ns*). We repeated this analysis for career self-management behavior. Again, no significant indirect effect was found (*b* = −0.01, 95% CI: −0.05 to 0.04, *ns*). This suggests that the intervention did not, directly or indirectly, significantly enhance physicians' perceptions of their employability (hypotheses 3a, 3b, and 3c rejected).

## Discussion

This intervention study tested and showed support that a newly developed career crafting training enhances physicians' perceptions of their job crafting behavior to decrease hindering job demands and their career self-management behavior. This is highly relevant today, given the diversity in career needs (Demerouti, 2014) and the demanding and changing career landscape (Frenk et al., 2010; Van den Heuvel et al., 2015). We did not find an impact of the training on perceptions of other types of job crafting and employability.

### Training and Job Crafting

The intervention enhanced physicians' perceptions of job crafting behavior to decrease hindering job demands. This was also the area where the most could be gained, due to the lowest score at T1. We did find different outcomes for various dimensions of job crafting, which is in line with previous studies on job crafting. For instance, Oprea et al. (2019) showed in a meta-analysis that the effects of job crafting interventions on various dimensions of job crafting differed. This variance may be caused by the variety in occupations that are studied. Different occupations may rely on specific combinations of job demands and resources, as argued in the job demands-resources model (Bakker and Demerouti, 2014). Therefore, various types of job crafting behavior may be performed differently across occupational groups (Nielsen and Abildgaard, 2012).

The present study showed that for physicians, actions to make their work less hindering were more common than adjusting work to make it more interesting or fit their strengths. This is in line with social-cognitive theory (Bandura, 1991) and the theory of planned behavior (Ajzen, 1991). Following Bandura's (1991) social-cognitive theory, physicians who participated in the career crafting training might have developed intentions to change their behavior. Some of these intentions have translated into actual behavior, while others have not. The theory of planned behavior proposes that intentions are more likely to translate into action if someone holds a favorable attitude toward these intentions. It could be that physicians were more motivated to engage in job crafting behavior to decrease hindering job demands than in other types of job crafting, e.g., to make their job more interesting. Examination of the intervention process revealed that this is in line with physicians' conversations in the group training. They talked very passionately about their job and were often proud of what they had accomplished. However, they were negative about time spent on administrative tasks and meetings. Other studies provide a similar picture, showing that while physicians are highly engaged (Smulders, 2006), they are frustrated about the time they must spend on hindering job demands such as administrative tasks and meetings (e.g., Rao et al., 2017). This suggests that physicians might already be satisfied with their job and may therefore invest less time in actions to make their work even more interesting, as compared to reducing the hindering aspects of their work.

### Training and Career Self-Management

The intervention enhanced physicians' perceived career self-management behavior. From previous studies we know that physicians do not usually take the time to reflect on their careers or on proactively managing their career (Löyttyniemi, 2001; Antoniou et al., 2003; Borges et al., 2010; Kragten, 2016). This view is in line with the conversation among physicians during the training. They mentioned that they were mainly working in the moment and did not take time to reflect on their future careers. Although physicians are not likely to invest in career self-management, this intervention study shows that this behavior can be trained and activated. The increase in physicians' career self-management behavior could be related to the assignment that they were asked to fulfill right after the training, working on self-set career crafting goals, and could be enhanced by the telephone consultation. Future research could examine whether this effect will last in the long run.

Physicians with different ages or in different career stages may engage in different types of job crafting and career self-management behaviors (Kooij et al., 2017). In this study, we did not examine possible differences between these groups. Rather, we chose to develop a training program for physicians of varying age and in various career stages to create diverse groups in order to facilitate peer-learning. Physicians' career experiences, challenges, and preferences may vary based on their age or experience. For instance, challenges around creating a healthy work–life balance might be more important for physicians with young children than for late-career physicians, and challenges about staying physically able to continue to work may be more common among late-career physicians. Future studies could examine whether outcomes of an intervention study like the one examined in this study would differ for physicians with different ages and experiences or study what would happen if such a training program would be offered to either early-, or mid-, or late-career physicians.

### Training and Employability

We did not find support for direct or indirect relationships between the intervention and physicians' employability. The examination of the intervention process showed that there was little variety in physicians' opinion about the training, as they were in general very positive. Therefore, it is unlikely that physicians' satisfaction with the training or atmosphere explains the lack of significant results for employability.

This lack of results for employability is more likely to be due to the content of the training and the type of outcome measures. The career crafting training was mainly designed to learn about, reflect on, and practice with career crafting behavior, instead of employability. Career crafting was therefore a proximal outcome referring to concrete behavior, while employability is a distal outcome that refers to an attitude instead of behavior. The fact that employability refers to a situation in the future might make it more difficult for physicians to perceive changes in their employability within a time period of 8 weeks. Employability might be subject to a so-called “sleeper effect,” which implies that the effect of the intervention on employability might appear not directly, but only after a period of time after exposure to the intervention (Frese and Zapf, 1988). Thus, physicians might have needed more time to work on their self-set goals in order to perceive an increase in their employability. Future studies could shed more light on this issue by including a long-term follow-up measurement in their design.

Physicians who participated in this study either worked in an academic or general hospital. Careers for these physicians can vary as physicians in academic hospitals can fulfill more diverse tasks (e.g., research and educational activities besides clinical tasks) than physicians in general hospitals. Therefore, we examined *post-hoc* whether the outcomes of any of the study variables differ for physicians working in the academic hospital and physicians working in the general hospital; no significant differences were found. In other words, the career crafting intervention works similarly for physicians in both hospitals and is relevant for physicians in both types of hospitals.

### Limitations

This is the first study to examine career crafting empirically. Since this is a relatively new field of research, one important limitation of this study is that we could not draw on a single, well-established conceptualization of career crafting; rather, several slightly different conceptualizations exist (e.g., De Vos et al., 2019; Tims and Akkermans, 2020). In conceptualizing and measuring career crafting, we therefore built on the few existing studies on career crafting (Akkermans and Tims, 2017; De Vos et al., 2019; Tims and Akkermans, 2020) and the established literature on proactive behaviors (i.e., job crafting and proactive career behaviors). Although we believe that our conceptualization and measurement of career crafting is a reasonable approximation of this concept, future studies could further examine, both theoretically and empirically, how career crafting can be enhanced and measured. One interesting proposal in this respect is the recently developed career crafting scale (Tims and Akkermans, 2020). Unfortunately, this instrument could not be used in this study since the intervention was developed before this scale was available.

Second, the intervention study design, although being important to study causality, came with the problem of non-compliance and missing data (Gupta, 2011). Some physicians did not complete the pre- or post-test, and some were not able to join the training session on the given date, resulting in non-compliance with the randomization in this study. As a result, the final sizes of the control and intervention group fell below the numbers of participants that were necessary to detect possible effects according to the a priori power calculation. However, the significant outcomes found in this study indicate that, *post-hoc*, we had enough power to detect at least some effects. A larger sample would perhaps have resulted in more significant outcomes, i.e., the number of significant effects reported here is possibly an underestimation of what would have been obtained when using a larger sample. Interestingly, only physicians who were originally assigned to the intervention group did not adhere to the allocated training moment, while all physicians in the control group complied with the study protocol. Physicians in the intervention group received the training 3–5 months earlier than physicians in the waitlist control group. This may have created scheduling conflicts, since most physicians have planning horizons of 3 months, while the dates for the training were announced 2 months before the start of the training sessions for the intervention group. Our specific interest in this study was to understand the effect of following a training program, instead of the effect of offering participants a training. Therefore, we did not perform intention-to-treat analyses but examined the 10 participants that were allowed to switch training dates, and subsequently moved from the intervention to the control group, within the control group. In doing so, we follow the approach of Kompier and Kristensen (2005), as we were interested in the effects of people actually following the training program. Several analyses were done to examine if this affected the randomization. No significant effects were found, showing that physicians in the waitlist control and intervention group do not differ on any of the study control variables.

Finally, in terms of the intervention design, due to the high-pressure work context, we had to use a brief intervention consisting of a single session, one follow-up phone call, and one follow-up measurement after 8 weeks to prevent dropout. As a result, we could only draw conclusions about the short-term effects of this training program. It remains unclear for how long these effects will last. However, as significant outcomes were found, it is likely that the impact of the intervention on relevant outcomes would even be stronger if more elaborate intervention designs are used. Furthermore, the most appropriate time frame between pre- and post-test is a topic of discussion. In the context of detecting job crafting effects, studies have shown that some job crafting behaviors have immediate effects, while others need more time to materialize (Tims and Bakker, 2010; Nielsen and Abildgaard, 2012). Kirkpatrick and Kirkpatrick (2016) developed a theory in which they argue that more time is needed for the effects of a training to be translated into actual behavior, than observing effects of a training on participants' level of satisfaction with it. We therefore consider 8 weeks appropriate to find effects on behavior. Future studies could further investigate the effects of more and longer follow-up sessions and could add another follow-up measurement to their designs to examine whether changes in employability need more time to be detected.

### Practical Implications

This study contributes to the literature on job crafting and career behavior since this is the first intervention study that examines career crafting empirically. An intervention study design is used, in which development is described in Van Leeuwen et al. (2020). This study shows how job crafting and career behavior can be addressed and stimulated in an intervention.

This study is also relevant for practice. Although the effects reported in this study were relatively small, they were statistically significant, showing that the intervention did increase the pro-active career crafting behaviors of physicians. Moreover, note that the effect sizes for especially career self-management behaviors and job crafting directed toward decreasing hindering job demands were in the area of 0.5–0.6 (i.e., a medium effect size, Cohen, 1988). This suggests that our findings not only were statistically significant but also will be practically relevant, and perhaps even more so when embedded in a broader training program or if accompanied with follow-up training sessions in which the learnt behaviors are refreshed and evaluated. This is particularly relevant to increase the likeliness that participants also engage in career crafting behavior in the long term, and this may perhaps also enhance their employability. HR managers can, for instance, use this study as an example to develop and implement a similar training program to enhance employees' proactive behavior. Moreover, the insights of this study can be used to develop and implement tailored career policies and career development practices. Although previous studies have shown that physicians hardly engage in behavior that prepares them for their further career (Löyttyniemi, 2001; Borges et al., 2010), this study shows that this behavior can be stimulated in an intervention. This is likely to be particularly relevant when a training program is tailored to the needs of the occupational group by involving intended users of the training in designing the training program.

## Conclusion

Overall, this career crafting intervention shows promising results for enhancing career crafting in physicians. Specifically, it was effective in enhancing job crafting to decrease hindering job demands and career self-management. This study contributes to the literature on career crafting, as this is the first test of an intervention study that examines how career crafting behavior can be enhanced in a training. No support was found for the effect of the intervention on employability. The insights may facilitate practical initiatives to encourage physicians' proactivity in making congruent choices about their job and career design.

## Data Availability Statement

The raw data supporting the conclusions of this article will be made available by the authors, without undue reservation.

## Ethics Statement

Ethical review and approval was not required for the study on human participants in accordance with the local legislation and institutional requirements. The patients/participants provided their written informed consent to participate in this study.

## Author Contributions

EL coordinated the study and gathered participants. EL drafted the manuscript. EK, TT, and J-WL secured funding for the project. MH designed the training content, and conducted the intervention together with EL. All authors conceptualized the research project, reviewed and provided comments, revisions, read, and approved the final manuscript.

## Conflict of Interest

The authors declare that the research was conducted in the absence of any commercial or financial relationships that could be construed as a potential conflict of interest.

## Footnotes

^1^The analyses were re-examined excluding these 10 participants. The outcomes were very similar to the ones presented in this article. The Time × Group interaction effect was not significant anymore (*p* = 0.076) in the RM ANOVA for job crafting behavior. Outcomes of the RM ANOVA analyses for the separate job crafting dimensions were similar. Moreover, the analyses were repeated where these 10 physicians were examined in the original group that they were assigned to (intention-to-treat analysis). There were some differences in the RM ANOVAs between the groups. For job crafting, the Time × Group (*p* = 0.189) and the Time × Type interaction effect (*p* = 0.067) were no longer significant. For career self-management, the Time × Group interaction effect was no longer significant (*p* = 0.091). The other main and interaction effects showed the same pattern of significance. More specific analyses that compared the outcomes on T1 and T2 within the control and within the intervention group resulted in the same pattern of significance.
